# Development of a Novel Ulcerative Colitis Endoscopic Mayo Score Prediction Model Using Machine Learning

**DOI:** 10.1016/j.gastha.2023.06.003

**Published:** 2023-06-17

**Authors:** David T. Rubin, Klaus Gottlieb, Jean-Frederic Colombel, Jean-Pierre Schott, Lavi Erisson, Bill Prucka, Sloane Allebes Phillips, John Kwon, Jonathan Ng, James McGill

**Affiliations:** 1 University of Chicago Medicine Inflammatory Bowel Disease Center, Gastroenterology, Chicago, Illinois; 2 Eli Lilly and Company, Immunology, Indianapolis, Indiana; 3 Icahn School of Medicine at Mount Sinai, Gastroenterology, New York, New York; 4 Iterative Scopes, Inc., Cambridge, Massachusetts; 5 KelaHealth, Inc., San Francisco, California; 6 Gensaic, Inc., Cambridge, Massachusetts; 7 Eli Lilly and Company, Advanced Analytics and Data Sciences, Indianapolis, Indiana; 8 Janssen Pharmaceuticals, Immunology, Raritan, New Jersey

**Keywords:** Inflammatory Bowel Disease, Central Reading, Artificial Intelligence, Deep Supervision

## Abstract

**Background and Aims:**

Endoscopic assessment is a co-primary end point in inflammatory bowel disease registration trials, yet it is subject to inter- and intraobserver variability. We present an original machine learning approach to Endoscopic Mayo Score (eMS) prediction in ulcerative colitis and report the model’s performance in differentiating key levels of endoscopic disease activity on full-length procedure videos.

**Methods:**

Seven hundred ninety-three full-length videos with centrally-read eMS were obtained from 249 patients with ulcerative colitis, who participated in a phase II trial evaluating mirikizumab (NCT02589665). A video annotation approach was established to extract mucosal features and associated eMS classification labels from each video to be used as inputs for model training. The primary objective of the model was a categorical prediction of inactive vs active endoscopic disease evaluated against 2 independent test sets: a full set with a baseline single human expert read and a consensus subset in which 2 human reads agreed.

**Results:**

On the full test set of 147 videos, the model predicted inactive vs active endoscopic disease via the eMS with an area under the curve of 89%, accuracy of 84%, positive predictive value of 80%, and negative predictive value of 85%. In the consensus test set of 94 videos, the model predicted inactive vs active endoscopic disease with an area under the curve of 92%, accuracy of 89%, positive predictive value of 87%, and negative predictive value of 90%.

**Conclusion:**

We have demonstrated that this machine learning model supervised by mucosal features and eMS video annotations accurately differentiates key levels of endoscopic disease activity.

## Introduction

Crohn’s disease and ulcerative colitis (UC), collectively called inflammatory bowel diseases (IBDs), are immune-mediated conditions characterized by chronic inflammation of the intestines and often extraintestinal manifestations.^1^^,^^2^ The degree of endoscopic and histologic inflammation of the bowel is associated with clinical severity and conversely, the absence of such inflammation (with healing) is associated with improved outcomes and durable disease control.^2^ An international consensus statement on treatment targets endorses an intermediate target of IBD therapy to be normalization of biochemical markers of inflammation and endoscopic healing and clinical remission as a longer-term target.^3^

The assessment of the degree of mucosal inflammation by endoscopy has become a critical part of disease assessment and response to therapy in clinical trials. A modern coprimary end point in IBD registration trials is the combination of both endoscopic healing and symptomatic remission.^4^^,^^5^ In registry trials for UC, the Endoscopic Mayo Sscore (eMS) is used to assess endoscopic disease activity. However, endoscopic assessment is subject to several challenges, including high inter- and intraobserver variability rates between human central readers,^6^^,^^7^ and a repeatedly demonstrated disconnect between clinical remission and endoscopic healing, which may be related to this variability as well as variable definitions of endoscopic improvement, remission, and mucosal healing.^8^ Such variability can increase the sample size required to demonstrate a significant change between active drug and placebo.^9^^,^^10^ Consequently, the Food and Drug Administration now requires phase III trials to apply adjudicated central reading, with a 2 + 1 central reading model (2 independent readers and one “tie breaker”) being the common method to improve the reliability of the score.^4^ However, this process is time consuming in general, and increasing the number of central readers specifically contributes to increased clinical trial costs and workflow delays.^10^

A potential solution is the use of machine learning (ML)^11^ and more specifically, deep learning models that can be trained to learn the mucosal features that drive human endoscopic disease activity interpretation. Prior studies have shown that deep learning models can produce eMS predictions from full-length endoscopy videos, albeit in a “black-box” fashion.^12^ An inherent ML problem of black-box models is that they do not provide visibility to the inner workings that produce the prediction outputs.^13^ In contrast to black-box models, deep supervision models use highly annotated datasets to fine-tune the model, which provides visibility to the features that influence the model’s prediction outputs.^14^ Another limitation of prior studies is that they have only compared eMS prediction outputs against a single human read,^12^ yet it is known that variability in human endoscopic interpretation affects the reliability of this model evaluation approach.

Here we present a novel ML approach for UC endoscopic disease activity prediction via the eMS and supervised through highly annotated endoscopic videos. We evaluated our model against 2 test sets: a full test set scored by a single human central reader and a consensus subset in which the 2 human readers agreed.

## Materials and Methods

The study objective was to develop a ML model that automates eMS prediction in UC from full-length endoscopic video recordings. Although the dataset provided both eMS and ulcerative colitis endoscopic index of severity scores, we chose to focus on eMS due to its wide application in clinical trials and the future potential to test on other datasets with historic eMS scores. We prospectively identified an accuracy (Acc) of 85% as the target model performance for the prediction of inactive (eMS = 0, 1) vs active (eMS = 2, 3) endoscopic disease, which we then evaluated against 2 independent test sets. This end point was selected based on recent evidence in image based scoring which supports expert review on image based MES scoring to be 82.8% (k = 0.78). In such assessments expert agreement with automated MES scoring was found to be 57.1% (k = 0.59) with an improvement to 69.5% when adjusted for reader variability.^15^

### Data source

A total of 793 standard-definition videos from 249 patients were de`identified and sourced from the phase II trial evaluating the efficacy of the p19 inhibitor mirikizumab in patients with moderately to severely active UC [Eli Lilly, Indianapolis, IN; NCT02589665].^16^ Trial enrollment occurred across 14 countries between January 2016 and September 2017, with study completion dated to May 2019. The 0-, 12-, and 52-week flexible sigmoidoscopy and full colonoscopy video recordings from this study were used for this project. Procedures were completed using late model Olympus or Fuji/Pentax equipment.^16^

A total Mayo score of 6–12 with an eMS ≥2, within 14 days before the first dose of study treatment was used to define a population that had moderately to severely active disease. All videos had a paired eMS scored by a single human central reader (Central Read Endoscopic Mayo Score [CReMS]). The videos provided had an endoscopic disease severity ranging from eMS = 0 to eMS = 3. As would be expected, the video dataset was weighted towards cases of active disease, with 76.9% of videos being an eMS = 2 or eMS = 3 case; eMS = 0 and eMS = 1 videos occurred less frequently, making up 6.4% and 16.6% of the dataset, respectively.

### Video data preprocessing

In order to develop the inputs for training the eMS prediction model, endoscopic features of disease and associated eMS labels were extracted from each video using a classification annotation approach. Classification annotation explicitly noted the presence or absence of mucosal features which could be used to train the model to learn the presence or absence of these features and to associate them to eMS classifications. Annotation was performed at a “clip” level; clips were defined as subsets of the video that have similar frames and can receive the same classification label.

Our annotation workforce produced over 100,000 clip-level labels, with over 60% of the labels related to annotation of specific features or eMS grades. The features include various subsets of *mild and marked erythema*, *decreased and absent vascular patterns*, *erosions*, *ulcerations*, *friability*, and *spontaneous bleeding*. The other 40% of the labels were constituted of frames which were part of clips labeled as eMS = 0 or were uninformative for the purposes of providing an eMS output, and these were therefore used as negative data to train the model to classify the absence of relevant features. These labels were also used to train a filter model to remove uninformative frames, which included those frames that were taken under water (or other fluid), contained poor bowel preparation, were blurry, were taken too close to the colon or rectal wall, were taken outside of the procedure, had suboptimal lighting, were completely black, or had endoscopic tools (forceps, etc) in the frames.

**Figure 1:**
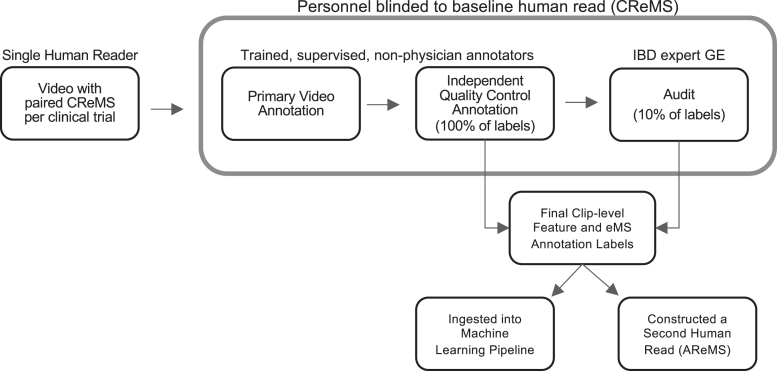
Overview of the video annotation workflow. The original dataset with full-length UC endoscopy video recordings was processed through a blinded annotation workflow. The annotation workflow consists of an initial video annotation, followed by a QC review that was performed by a second annotator on 100% of the labels; 10% of labels were audited by an IBD expert GE. Annotation classification labels were ingested into the ML pipeline and used to construct a second baseline human read eMS (AReMS). AReMS, Annotator Reported Endoscopic Mayo Score; CReMS, Central Read Endoscopic Mayo Score; eMS, Endoscopic Mayo Score; GE, gastroenterologist; IBD, inflammatory bowel disease.

The annotation workflow consisted of 3 distinct steps: primary annotation, quality control (QC) annotation, and audit, as shown in Figure 1. Primary and QC annotations were performed by trained, supervised, nonphysician annotators. QC was performed by a second annotator in 100% of the labels generated by the primary annotator. An audit by an IBD expert gastroenterologist (GE) was performed in cases where the first and second annotator disagreed. In addition, 10% of all annotations were randomly sampled and audited. IBD expert gastroenterologists were defined as GEs who had completed an IBD fellowship and had previous experience as a central reader for clinical trials in UC. GE review approval rates were used to retrain annotators, which proved effective in increasing GE approval rates.

Throughout the annotation workflow, all personnel were blinded to the clinical trial’s CReMS, which prevented annotator bias and allowed for an independent evaluation of endoscopic disease activity in each video. Clip-level annotations resulting from the annotation workflow were used to construct an overall video-level eMS score, independent of the CReMS, referred to as the Annotator Reported Endoscopic Mayo Score (AReMS). Once videos completed the annotation workflow, they were ingested into the ML pipeline.

### Data sampling approach

**Figure 2:**
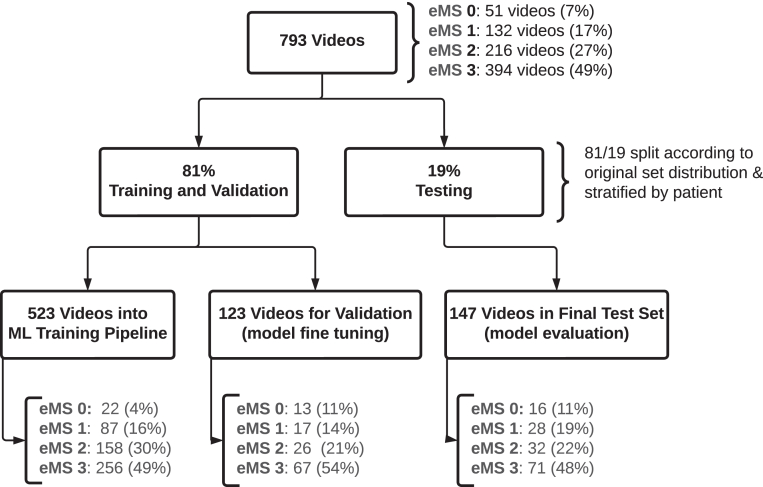
Overview of the data sampling approach including model training, validation, and evaluation sets. The standard 80/20 split was used and the distribution of eMS classes within each subset was maintained from the original dataset and is shown. eMS, Endoscopic Mayo Score; ML, machine learning.

In order to train and evaluate the performance of the ML model, we created training, validation, and testing datasets according to dataset creation in ML standard practices^17^; 81% of the videos were used for training and validation, the remaining 19% were used for independent model testing, as shown in Figure 2. All the datasets were stratified by patient to ensure that the same patient would not be represented simultaneously in both training and test sets, which could lead to bias in the testing phase of model development. These splits were maintained for all model training experiments. The original distribution of eMS classes was maintained across the datasets. For cross-validation experiments, the training and validation sets were combined, generating 5 splits at random, stratified by patient.

### Model training and validation networks

Feature and eMS classification annotations were used as inputs to train the eMS prediction model. Model validation involved learning model parameters and tuning of hyperparameters such as learning rate and loss function in order to minimize differences between annotation labels and model prediction outputs.

**Figure 3:**
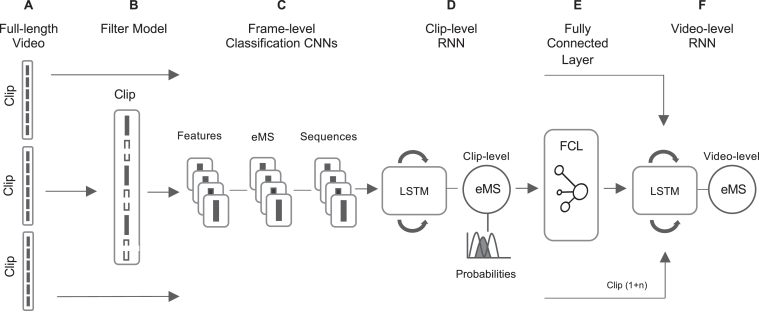
Overview of the end-to-end ML Process. (A) Full-length videos were decomposed into fixed-length clips; (B) the filter model downsampled uninformative frames; (C) frame-level feature and eMS classification convolutional neural networks outputted frame-level sequences; (D) the clip-level recurrent neural network (long short-term memory) outputs a clip-level eMS prediction and the associated probabilities; (E) the fully connected layer identified the most significant outputs; (F) the video-level long short-term memory distilled all video-clip outputs into a full-length video eMS. Clip (1 + n) indicates that there were more than 3 clips in the end-to-end processes that fed the final video-level eMS. CNN, convolutional neural network; eMS, Endoscopic Mayo Score; FCL, fully connected layer; LSTM, long short-term memory; RNN, recurrent neural network.

The end-to-end ML process is represented in Figure 3. Full-length videos were decomposed into clips and frames. A filter model downsampled images by removing uninformative frames prior to passing them to the frame-level classification models. Feature and eMS classification convolutional neural networks were used to explicitly classify the presence or absence of relevant features, and to generate feature to eMS association sequences based on individual frames. The eMS classification of video-clips and full-length video feeds were built on the foundation of these frame-level models, which generated eMS predictions using different sequence-based long short-term memory architectures that accept frame classification sequences as inputs and then outputted predicted clip-level eMS predictions and its associated probabilities. A fully connected layer then identified the most significant clip-level outputs that should contribute to a video-level eMS. Finally, a second long short-term memory aggregated all sampled clip-level eMS outputs to produce a full-length video eMS.

### Model performance evaluation

We evaluated the model’s ability to predict endoscopic disease activity on full-length videos against 2 independent test sets which were previously unseen by the model. On the full test set, performance was evaluated against a single human CReMS obtained via the clinical trial design. The annotation workflow produced a second human read, the AReMS, which enabled comparison of 2 independent human reads. Using the subset of videos from the full test set in which the CReMS and AReMS agreed, we derived a consensus test set. We evaluated the study endpoints against the full test set and the consensus test set.

The primary end point evaluated the model’s performance in prediction of inactive (eMS = 0,1) vs active (eMS = 2,3) endoscopic disease. The secondary endpoints evaluated model performance in prediction of endoscopic healing (eMS = 0 vs eMS = 1, 2, 3), as well as prediction of severe endoscopic disease (eMS = 3 vs eMS = 0, 1, 2).

Receiver operating characteristic curves and confusion matrices were used to visualize model performance in a binary classification. The study end points were evaluated using area under the curve (AUC), Acc, positive predictive value (PPV), and negative predictive value (NPV).

## Results

### Human reader agreement

**Figure 4:**
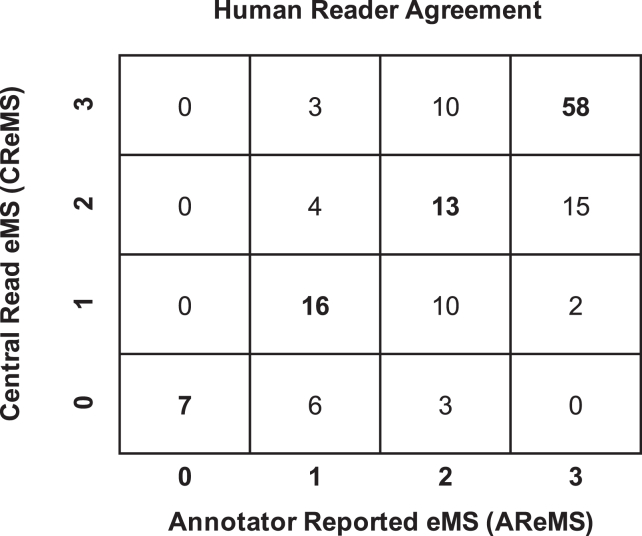
Four × 4 confusion matrix showing the overlap between the CReMS and the AReMS baseline human assessment in 147 videos of the full test set. The diagonal bold numbers indicate those videos where the CReMS and the AReMS were in agreement, which comprises the consensus test set.

eMS agreement on the video-level baseline human score between the CReMS and AReMS in the full test set of 147 videos is shown in Figure 4. Ninety-four videos from the original test set were found to have a consensus eMS, with an overall agreement rate of 64%. The full and consensus test sets were both weighted toward active disease, with 70% and 76% of the videos classified as eMS = 2 or 3, respectively. The CReMS and AReMS had a higher agreement for severe endoscopic disease (eMS = 3) than the other eMS classes.

### Primary end points

**Figure 5:**
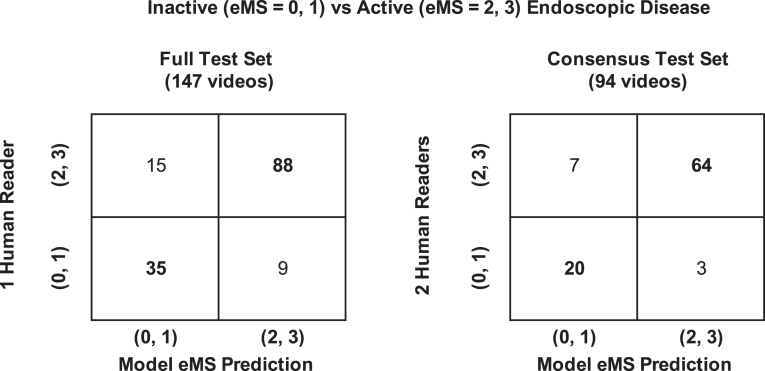
Two × 2 confusion matrices of model eMS predictions tested against human-reads of the full (147 videos) and consensus (94 videos) test sets. The numbers within each quadrant refer to the total number of videos that fall into a grouped classification of inactive (eMS = 0, 1) or active (eMS = 2, 3) endoscopic disease comparing the baseline human score (y-axis) and the model’s eMS prediction (x-axis). Model performance accuracy metrics (Acc, PPV, NPV) can be derived from these confusion matrices. eMS, Endoscopic Mayo Score.

On the full test set of 147 videos, the model predicted inactive vs active endoscopic disease with an AUC of 89%, Acc of 84%, PPV of 80%, and NPV of 85%. On the consensus test set of 94 videos, the model predicted inactive vs active endoscopic disease with an AUC of 92%, Acc of 89%, PPV of 87%, and NPV of 90% (Appendix 1). Two 2 × 2 confusion matrices comparing the model’s predictions to the full test set and the consensus test set are shown in Figure 5. Across all primary endpoint metrics, performance of the model was higher on the consensus test set.

### Secondary end points

The model predicted endoscopic healing on the full test set with an AUC of 88%, Acc of 90%, PPV of 44%, and NPV of 95%; on the consensus set, the model predicted endoscopic healing with an AUC of 95%, Acc of 95%, PPV of 86%, and NPV of 95%.

The model predicted severe endoscopic disease on the full test set with an AUC of 85%, Acc of 80%, PPV of 72%, and NPV of 87%; on the consensus set, the model predicted severe endoscopic disease with an AUC of 87%, Acc of 85%, PPV of 78%, and NPV of 90% (Appendix 2).

**Figure 6:**
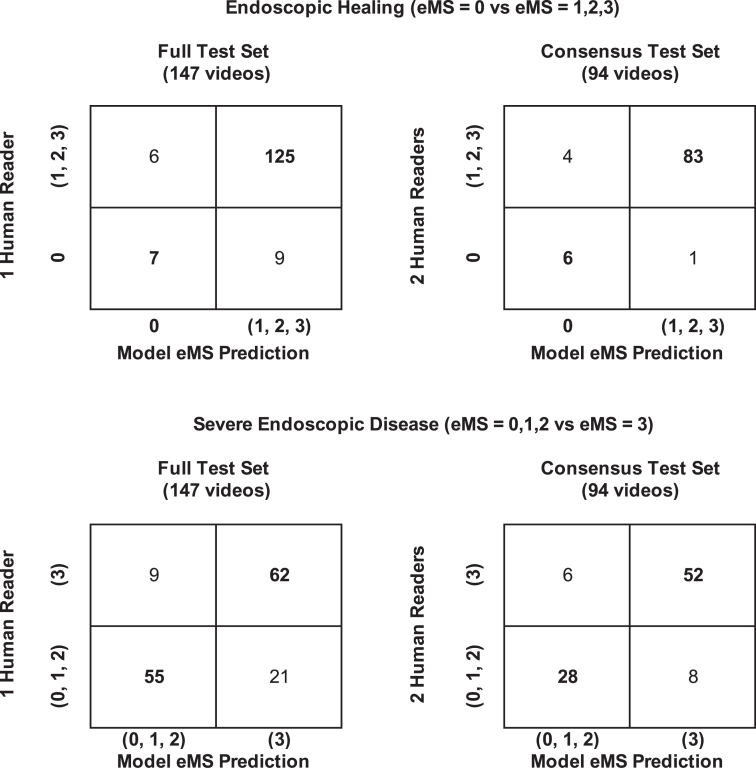
Two × 2 confusion matrices of model eMS prediction tested against human-reads of the full (147 videos) and consensus (94 videos) test sets. The model is able to distinguish endoscopic healing and severe endoscopic disease. eMS, Endoscopic Mayo Score.

The model’s performance for prediction of endoscopic healing and severe disease is presented in Figure 6, with the 2 2 × 2 confusion matrices comparing the model’s predictions against the human eMS reads on the full and consensus test sets.

## Discussion

We have developed a novel ML model that was trained on independently annotated full-length endoscopy videos from patients with UC which predicts the eMS. The model’s performance was evaluated against a unique test set and demonstrated an excellent distinction between inactive and active endoscopic disease as well as a clear discrimination between endoscopic healing and severe endoscopic disease.

Our model training and evaluation approach addresses the limitations of previous deep learning models. First, prior attempts at using deep learning models to predict the endoscopic disease activity in UC relied on black-box approaches; while a human baseline eMS was available, no human video annotation work for classification of mucosal features and associated eMS was done. Given the paucity of central human readers in a given clinical trial, black-box approaches also risk modeling individual readers, which may not be generalized. While time consuming, we believe that the use of well-trained, narrowly focused, nonphysician annotators, who are subjected to rigorous quality review and who also fulfill annotation Acc requirements, is a crucial element of future computer vision aided clinical research projects, in IBD and elsewhere.

Second, the performance of deep-learning models inherits the cognitive limitations of the human reader data it was trained on and thus cannot outperform the human reader. As a consequence, these models may lack robustness to perform outside of their respective research samples. In contrast, our deep supervision model was fine-tuned through mucosal features and associated eMS classification labels, expanding on the ability of any individual human reader to fully identify every mucosal feature in an endoscopy procedure. Therefore, we believe this approach can become the steppingstone for a ML endoscopic activity score that can surpass the cognitive limitations of a human reader.

Lastly, we have been able to examine the quality of a single human reader eMS as the baseline score against which to evaluate eMS prediction models. In previously published studies, a single central read model has been the measurement used to evaluate model performance. In our study, a robust annotation pipeline produced a second and independent human read, which fed the creation of a unique evaluation set. The consensus test set represents the eMS interpretation agreement of 2 human reads; the metrics used to assess model performance improved in this test set of videos with a consensus eMS agreement.

The use of this eMS prediction model can reduce the variability in endoscopic disease assessment of clinical trial central reads, improving consistency and reproducibility. As previously mentioned, reducing variability in clinical trial end point measurements can have significant benefits for sample sizes and the associated statistical power required to differentiate active drugs from placebo. Furthermore, standardized, high quality eMS automation can reduce clinical trial screening failure rates by improving the endoscopic assessment of clinical trials’ patient eligibility criteria.^11^

The results of our study compare favorably with previous studies.^18^ To our knowledge, only Gottlieb et al^12^ have trained an eMS prediction model with full-length videos derived from a large UC clinical trial but utilized a different model training and evaluation approach. Appendix 3 juxtaposes our study results with this previous study, which used the same clinical trial videos and associated single human centrally-read eMS, but without the deep supervision provided by our annotation pipeline and without the human agreement consensus test set model evaluation approach. Nonetheless, both models achieved excellent Acc in prediction of endoscopic disease activity and endoscopic healing. The model developed by Gottlieb et al, achieved an endoscopic disease activity (eMS = 2, 3) prediction Acc of 92% and an endoscopic healing (eMS = 0) prediction Acc of 95%^12^; in our consensus test set, our model achieved a similarly excellent prediction Acc of 89% and 95%, respectively.

There are several limitations to our study. First, the dataset used in this study was weighted toward endoscopic moderately to severely active disease (76%), per the clinical trial design. Moreover, the same dataset that was used for training and validating the model was also used for model evaluation; and although the data sampling approach mitigates overfitting, generalizability will be tested on different clinical trials and real-world datasets in the future. Secondly, using a single human baseline eMS as a measurement to evaluate model performance did not enable us to establish robustness of the model in the presence of interobserver variability rates. While our annotation workflow and consensus test set provided the agreement of 2 human readers, no centrally-read adjudication was available in the full test set. Therefore, the consensus test set may have been biased to exclude controversial cases. More work is clearly needed, especially the development of ML models from large clinical trials that are using or have used the 2 + 1 central read model.

## Conclusion

In conclusion, we have demonstrated a UC eMS prediction model supervised through training on the key mucosal features that drive the eMS performs as well or better than black-box approaches. This is foundational for an endoscopic disease activity model that will surpass a human reader, and which we plan to integrate other data sources in order to achieve a disease progression model and a comprehensive total disease activity end point.

This novel ML approach to endoscopic disease assessment may eventually replace human central reading in clinical trials and will undoubtedly have significant benefits for standardization of interpretation and management decisions in clinical practice.
