# Supplementary material for: Development of a Novel Ulcerative Colitis Endoscopic Mayo Score Prediction Model Using Machine Learning
Source: Gastro Hep Adv. 2023 Jun 17;2(7):935–42. doi: 10.1016/j.gastha.2023.06.003 (PMC11307476; doi:10.1016/j.gastha.2023.06.003)
Supplement: Appendix 1–3 [file mmc1.docx]

Appendix

**Appendix 1. Primary Endpoint**

| **Primary endpoint  (eMS = 0,1 vs eMS = 2,3)** | **Full test set (147 videos)** | **Consensus test set (94 videos)** |
| --- | --- | --- |
| AUC | 89% | 92% |
| ACC | 84% | 89% |
| PPV | 80% | 87% |
| NPV | 85% | 90% |

AUC, area under the curve; ACC, accuracy; PPV, positive predictive value; NPV, negative predictive value

**Appendix 2. Secondary Endpoints**

| **Secondary endpoints** | **Key metrics** | **Full test set (147 videos)** | **Consensus test set**  **(94 videos)** |
| --- | --- | --- | --- |
| **Endoscopic healing**  (eMS = 0 vs 1,2,3) | AUC | 88% | 95% |
|  | ACC | 90% | 95% |
|  | PPV | 44% | 86% |
|  | NPV | 95% | 95% |
| **Severe Endoscopic Disease**  (eMS = 3 vs 0,1,2) | AUC | 85% | 87% |
|  | ACC | 80% | 85% |
|  | PPV | 72% | 78% |
|  | NPV | 87% | 90% |

AUC, area under the curve; ACC, accuracy; PPV, positive predictive value; NPV, negative predictive.

**Appendix 3. Categorical eMS Prediction Accuracy Comparison to Prior Study**

| **Study** | **# Videos in Evaluation Set** | **# Reviewers** | **Inactive vs Active Endoscopic Disease (Accuracy)** | **Endoscopic Healing (Accuracy)** |
| --- | --- | --- | --- | --- |
| **Gottlieb, et al.**^12^ | 134 | 1 | 92% | 95% |
| **Current Study** -  Full set | 147 | 1 | 84% | 90% |
| **Current Study** - Consensus set | 94 | 2 | 89% | 95% |

Appendix 3 juxtaposes the accuracy results of this study with a previous study that utilized the same full-length video data source for model training and validation. Our model has similarly excellent performance than this black-box approach, particularly in the consensus set.
